# Enhanced Response of T Cells from Murine Gammaherpesvirus 68-Infected Mice Lacking the Suppressor of T Cell Receptor Signaling Molecules Sts-1 and Sts-2

**DOI:** 10.1371/journal.pone.0090196

**Published:** 2014-02-28

**Authors:** Brandon Cieniewicz, Nicholas Carpino, Laurie T. Krug

**Affiliations:** Molecular and Cellular Biology Program and Department of Molecular Genetics and Microbiology, Stony Brook University, Stony Brook, New York, United States of America; Geisel School of Medicine at Dartmouth, United States of America

## Abstract

The human gammaherpesviruses establish life-long infections that are associated with the development of lymphomas and neoplasms, especially in immunocompromised individuals. T cells play a crucial role in the control of gammaherpesvirus infection through multiple functions, including the direct killing of infected cells, production of cytokines such as interferon-γ (IFN-γ), and costimulation of B cells. Impaired T cell function in mice infected with murine gammaherpesvirus 68 (MHV68) leads to increased reactivation and pathologies, including a higher incidence of lymphoid hyperplasia. Here we report that the absence of Suppressor of TCR signaling −1 and −2 (Sts-1^-/-^/2^-/-^) during MHV68 infection leads to the generation of T cells with significantly heightened responses. Transient differences in the T and B cell response of infected Sts-1^-/-^/2^-/-^ (Sts dKO) mice were also observed when compared to WT mice. However, these alterations in the immune response and the overall absence of Sts-1 and Sts-2 did not impact viral pathogenesis or lead to pathology. Acute lytic replication in the lungs, establishment of latency in the spleen and reactivation from latency in the spleen in the Sts dKO mice were comparable to WT mice. Our studies indicate that Sts-1 and Sts-2 are not required for the immune control of MHV68 in a normal course of gammaherpesvirus infection, but suggest that interference with negative regulators of T cell responses might be further explored as a safe and efficacious strategy to improve adoptive T cell therapy.

## Introduction

The human gammaherpesviruses Epstein-Barr virus (EBV/HHV-4) and Kaposi's Sarcoma-associated Herpesvirus (KSHV/HHV-8) collectively infect over 95% of individuals, causing life-long infections that predispose infected individuals to the development of malignancies [1]–[4]. While the extent of productive replication upon primary infection with EBV or KSHV is not clear, these viruses ultimately establish a latent infection wherein the genome is maintained, but few viral proteins are expressed [5]–[8]. In an immunocompetent host, immune surveillance by virus-specific T cells controls intermittent virus reactivation from latency [9]–[13]. However, loss of immune control increases the risk of malignancies in viral reservoirs including B lymphocytes (EBV and KSHV), epithelial cells (EBV) and endothelial cells (KSHV) [14], [15]. Reactivation and persistent infection cause disease in HIV-infected individuals (e.g Kaposi's Sarcoma), while the seeding of naïve lymphocytes leads to uncontrolled proliferative expansion in EBV- or KSHV-negative transplant recipients (e.g. post transplant lymphoproliferative disorder, PTLD) [16], [17].

The murine gammaherpesvirus 68 is a natural pathogen of murid rodents with genetic and biological similarities to the human gammaherpesviruses [5], [18]. This model pathogen has aided in the dissection of the roles of T lymphocytes during a natural host infection [19]–[21]. Both CD4^+^ and CD8^+^ T cells promote clearance of productive replication in the lung during acute infection [22], [23]. T cell surveillance plays a critical role in control of MHV68 during the chronic, latent phase of infection [9], [24], [25]. Virus specific CD8^+^ T cells persist for the life of the infected host [9], [11], [26], [27] and secrete effector molecules such as perforin and IFNγ that are necessary to repress reactivation from B cells and macrophages, respectively [24], [28], [29]. Activated CD4^+^ T cells are present throughout chronic infection to promote B cell responses, support CD8^+^ T cell effector function [30], [31], and directly inhibit reactivation through the secretion of cytokines [12], [13], [22], [32], [33]. T cells specific for viral antigens exposed during latency control virus expansion in the spleen, while those that recognize lytic epitopes prevent viral recrudescence in the lungs [9], [27], [32]. Vaccination that drives the generation of virus-specific CD8 T cells reduces viral burden [34], as does adoptive transfer of virus-specific T cells to naïve mice prior to infection [35].

Effective T cell function requires signaling through costimulatory receptors and sustained activation of intracellular signaling pathways. Alteration of these signaling factors can reduce or enhance T cell responses, which in turn impacts control of MHV68 latency. A knockout mouse lacking both B7-family receptors CD80 and CD86 has severe defects in IFNγ secretion by T cells and the response to secondary infection, in addition to a failure to produce neutralizing antibodies and maintain long-term control of latency [36], [37]. Deletion of another B7 family member, 4-1BB, impairs T cell effector function and leads to increased viral latency [38]. On the other hand, mice lacking the SLAM associated protein (SAP), a negative regulator of lymphocyte signaling, have increased CD8^+^ T cell activation in response to infection and impaired antibody production that ultimately does not alter long term control of the virus [39], [40]. The role of mutations that generate gain-of-function T cells in the absence of other off-target B cell effects in the control of MHV68 infection has not been determined.

Negative feedback molecules control the duration of T cell activation by engagement of inhibitory receptors or inactivation of TCR signaling intermediates through direct binding, phosphatase activity, or ubiquitin ligase activity [41]. Removal of these negative regulators can increase TCR sensitivity [42], extend the duration of T cell effector function [43] or increase effector function [41], [43]–[45]. Sts-1 and Sts-2 (TULA-2 and TULA-1, respectively) are intracellular phosphatases conserved in mice and humans that promote the dephosphorylation of the TCR signaling intermediates Zap-70 and syk [46]–[49]. Sts-1 is ubiquitously expressed, whereas Sts-2 has a more restricted pattern of expression, predominantly in naïve and mature T cells [46], [47]. Sts-1 and Sts-2 serve distinct but compensatory inhibitory roles in TCR signaling that impact T cell biology [47], [48]. Disruption of both Sts genes lowers the threshold of T cell activation and leads to increased cytokine production and proliferation in response to TCR stimulation [48]. In addition, loss of these TCR regulators confers increased incidence of autoimmunity in a model of multiple sclerosis [47].

In this study we examined the impact of Sts-1 and Sts-2 on the control of gammaherpesvirus pathogenesis. We infected Sts-1/2 double knockout mice (Sts dKO) with MHV68 and monitored immune responses and viral burden at multiple time points during the acute and chronic phases of infection. We found that the absence of the negative TCR regulators Sts-1 and Sts-2 led to increased effector T cell responses in culture that did not impact the normal course of MHV68 pathogenesis *in vivo*.

## Materials and Methods

### Ethics Statement

All of the animal experiments described herein were done under strict observance of the National Institute of Health guidelines. The Stony Brook University Institutional Animal Care and Use Committee approved this study (IACUC protocol #253637). All efforts were made to minimize suffering. Infection and adoptive T cell transfers were performed under isofluorane anesthesia.

### Mice

Mice bearing germline Sts-1^-/-^ and Sts-2^-/-^ (Sts dKO) on a C57BL/6 background were bred in our facility [47]. Age and sex matched C57BL/6 mice were purchased from Jackson Labs (Maine, USA). 8–12 week old animals of mixed genders were used in groups of 3–7 for most experiments. Experiments were conducted with BSL2 safety precautions.

### Cell Culture

Low passage murine embryonic fibroblasts were cultured at 37°C 5% CO_2_ in DMEM containing 10% FBS, 2 µM L-glutamine, 50 U/ml penicillin and 50 µg/ml streptomycin (10% CMEM). NIH murine 3T12 fibroblasts were cultured at 37°C 5% CO_2_ in DMEM containing 8% FBS, 2 µM L-glutamine, 50 U/ml penicillin and 50 µg/ml streptomycin (8% CMEM).

### Virus and Infections

For all infection experiments, we used the WT MHV68 isolate (WUMS strain, ATCC # VR1465) that was propagated as previously described [50]. For intranasal infection, mice were lightly anesthetized using isofluorane and infected with 1,000 plaque forming units (PFU) of virus in a 20 µl bolus of 10% CMEM applied to the nose. Back titers of inoculate were performed to confirm infectious dose. For intraperitoneal infections, mice were lightly anesthetized using isofluorane and injected intraperitoneally with 1000 PFU of virus in 500 µl of 10% CMEM. For infections of primary MEFs, semi-confluent MEFs were infected at a multiplicity of infection (MOI) of 10 PFU with WT MHV68 24 h prior to use. Peritoneal exudate cells were isolated by peritoneal injection of 10 ml of DMEM, agitating the abdomen, and withdrawing the peritoneal wash by syringe.

### Flow Cytometry

For analysis of immune cell responses in mouse tissues, 2×10^6^ cells per sample were resuspended in 200 µl PBS+2% FBS (FACS). Cells were blocked with TruStain fcX (Clone 93, Biolegend), washed, and stained with the following combinations: Effector T cell (CD4 or CD8, CD62L, CD44), Vβ4 T cells (CD3, CD8, Vβ4, CD62L), p79 tetramer CD8^+^ T cell (CD8, CD62L, p79 tetramer kindly provided by the NIH Tetramer facility, TSINFVKI/H-2K^b^), p56 tetramer CD8^+^ T cell (CD8, CD62L, p56 tetramer kindly provided by the NIH Tetramer facility, AGPHNDMEI/H-2K^b^), activated B cell (CD19, CD69), germinal center B Cell (CD19, CD95, GL7). Cells were analyzed using a FACScalibur or Dxp8 FACScan (Cytek Development; BD Biosciences).

For analysis of T cell effector responses, spleens were isolated from naïve and infected mice 28 dpi. 2×10^6^ bulk splenocytes were plated into each well of a 96-well round bottomed plate and either left untreated or treated with 1 µg/ml LEAF-purified αCD3 antibody (Clone 17A2, Biolegend), or 2×10^5^ 24 hr-infected MEFs that were gamma-irradiated with 2000 rads immediately prior to coculture. Cells were incubated overnight at 37°C for 12 h in CMEM with golgiplug (BD Biosciences) and LAMP1/CD104a antibody (Clone 1D4B, Biolegend). The Fc receptors were blocked (TruStain fcX Clone 93, Biolegend) prior to staining with the following antibodies: CD8 (Clone 53–6.7, Biolegend), CD19 (Clone 6D5, Biolegend), and CD3 (Clone 145-2C11, Biolegend). Cells were also stained with APC-conjugated p79 tetramer. Upon fixation and permeabilization with the BD Cytofix/Cytoperm kit (BD Biosciences), cells were stained with IFNγ (Clone XMG1.2, Biolegend). Cells were analyzed using a Dxp8-FACScan (Cytek Development; BD Biosciences) and data was analyzed using FlowJo vX (Treestar).

### Viral pathogenesis assays

For acute titers, mice were sacrificed by isofluorane at the indicated days post infection, the left lung was removed, frozen at −80°C, thawed, and disrupted in 1 ml of 8% CMEM using 1 mm beads in a bead beater (Biospec). Serial dilutions in CMEM were plated on subconfluent monolayers of NIH 3T12 murine fibroblasts in 6 well plates. Plates were rocked intermittently for 1 h and then overlaid with 3 ml of 8% CMEM+1.5% methylcellulose. Cells were fixed after 1 week, stained, and counted.

For quantitation of latency, limiting-dilution nested PCR with primers for the MHV68 ORF50 region was used to determine the frequency of virally infected cells. Frozen samples were thawed, resuspended in isotonic buffer, counted, and plated in serial 3-fold dilutions into a 96-well plate in a background of 10^4^ NIH 3T12 murine fibroblasts. Six serial dilutions were plated, and 12 wells were plated per dilution. Plates were covered with PCR foil (Eppendorf Scientific), and cells were lysed with proteinase K for 6 h at 56°C prior to the addition of 10 µl of round 1 PCR mix to each well by foil puncture. Following first-round PCR, 10 µl of round 2 PCR mix was added to each well by foil puncture and samples were subjected to round 2 PCR. Products were resolved on 2% agarose gels and each dilution was scored for positive bands. Control wells containing uninfected cells or 10, 1, and 0.1 plasmid copies of ORF50 were run with each plate to ensure single-copy sensitivity and no false positives. Data is representative of three independent experiments with four to six mice each. Error bars represent standard deviation.

For quantitation of reactivation, a limiting-dilution reactivation assay was performed. Bulk splenocytes in cMEM were plated in serial 2-fold dilutions (starting with 10^5^ cells) onto MEF monolayers in each well of a 96-well tissue culture plates. Twelve dilutions were plated per sample, and 24 wells were plated per dilution. Wells were scored for cytopathic effect at 21 d postplating. To detect preformed infectious virus, parallel samples of mechanically disrupted cells were plated onto MEF monolayers.

### Statistical Analyses

All data were analyzed by using GraphPad Prism software (GraphPad Software, http://www.graphpad.com). Titer data were analyzed for Gaussian distribution using the D'Agostino and Pearson omnibus normality test followed by an unpaired t-test or a non-parametric Mann-Whitney two-tailed *t*-test. The frequencies of reactivation and viral genome–positive cells were obtained from the nonlinear regression fit of the data where the regression line intersected 63.2% based on the Poisson distribution, and then statistically analyzed by unpaired two-tailed *t*-test.

## Results

### Sts dKO T cells have heightened responses to MHV68 infected cells in culture

**Figure 1:**
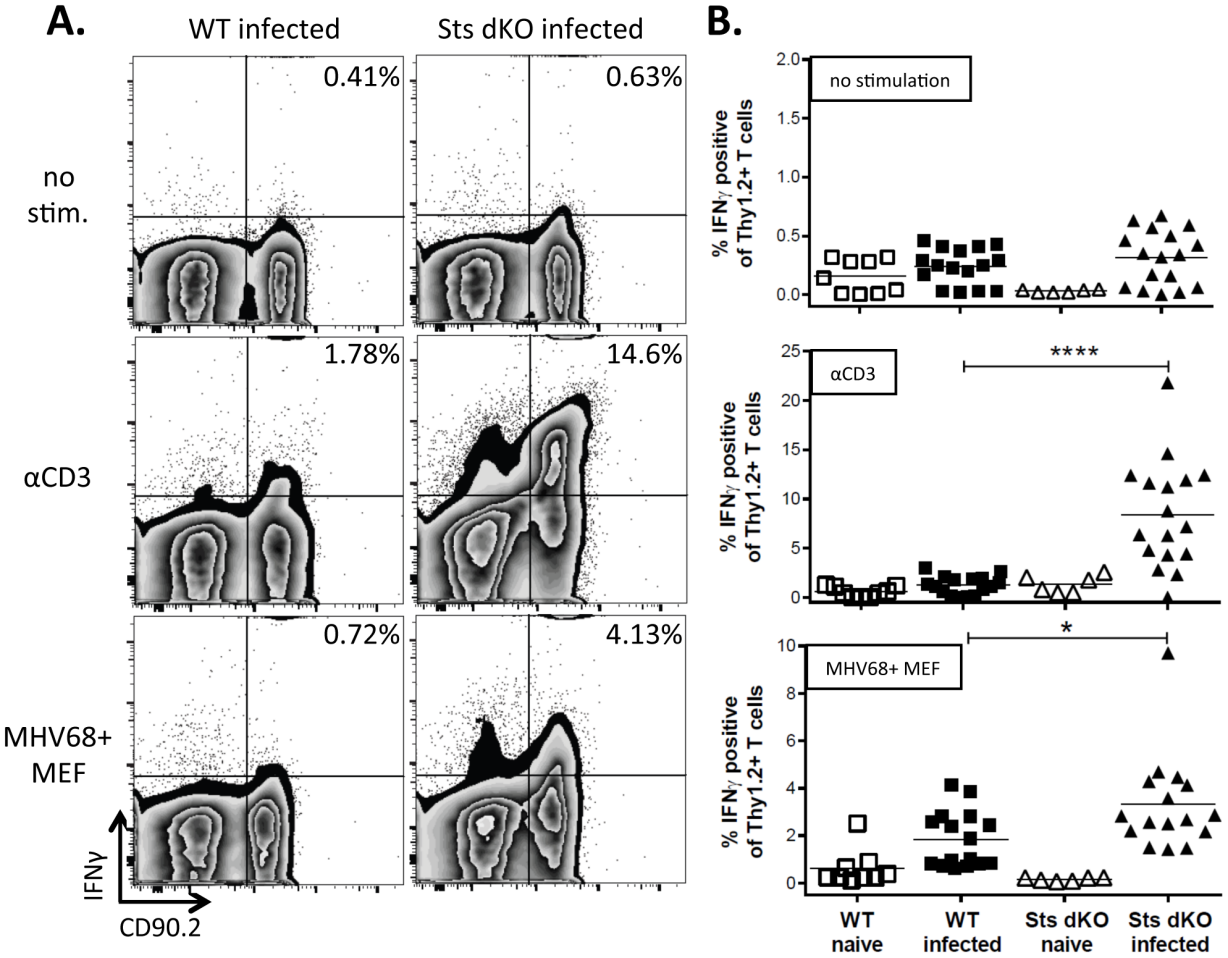
Increased IFNγ response to infected cells in the absence of Sts1 and Sts2. Sts dKO and C57BL/6 WT mice were infected 1000 PFU of MHV68 by the intranasal route and spleens were harvested 28 dpi. Splenocytes were left untreated or stimulated with 1 ug/ml αCD3 antibody or cocultured with gamma-irradiated MHV68-infected MEFs, both overnight in the presence of monensin. Cells were stained with the T cell marker CD90.2 and the intracellular cytokine IFNγ and analyzed by flow cytometry. (A) Representative flow plots of IFNγ expression in cells stained for CD90.2 are shown for each genotype and culture condition. (B) Scatter plot summary of the percentage of T cells producing IFNγ+ after overnight stimulation. Symbols represent data from individual mice. * = p<.05, ****  = p<.0001.

Mice that lack the antiviral cytokine IFNγ or the IFNγ receptor exhibit increased susceptibility to MHV68 infection, characterized by uncontrolled lytic infection, pathology-induced pneumonia, fibrosis of the lungs and spleen, and death [22], [23], [51]–[54]. A striking phenotype of Sts dKO mice is the production of hyperresponsive T cells that lead to enhanced proliferation and cytokine responses, including IFNγ upon TCR stimulation [47], [48], [55], [56]. We reasoned that the T cells from Sts dKO mice might be hyperresponsive to MHV68 infection. To examine the role of Sts-1 and Sts-2 in the T cell response to gammaherpesvirus infection, we infected Sts dKO and WT mice with WT MHV68 by the intranasal route of infection. Splenocytes isolated at 28 days post-infection (dpi) were simulated overnight with 1 µg/ml αCD3 antibody in the presence of monensin to capture intracellular IFNγ production. Unstimulated Sts dKO and WT T cells were largely IFNγ negative (Figure 1A, top). However, when stimulated with a low level of αCD3, T cells from infected mice were more sensitive to non-specific TCR stimulation than their naïve counterparts (Figure 1B, middle). The Sts dKO mice had a significant five-fold increase in the production of IFNγ by T cells as compared to WT mice (Figure 1A&B, middle). Similar to what we observed with non-specific antibody stimulation, there was a significant increase in the levels of IFNγ production in Sts dKO T cells relative to WT T cells following stimulation with gamma-irradiated murine embryonic fibroblasts (MEFs) infected with MHV68 (Figure 1A&B, bottom). This data indicates that Sts dKO T cells from infected mice have a heightened response to viral antigens as compared to T cells from infected WT mice.

**Figure 2:**
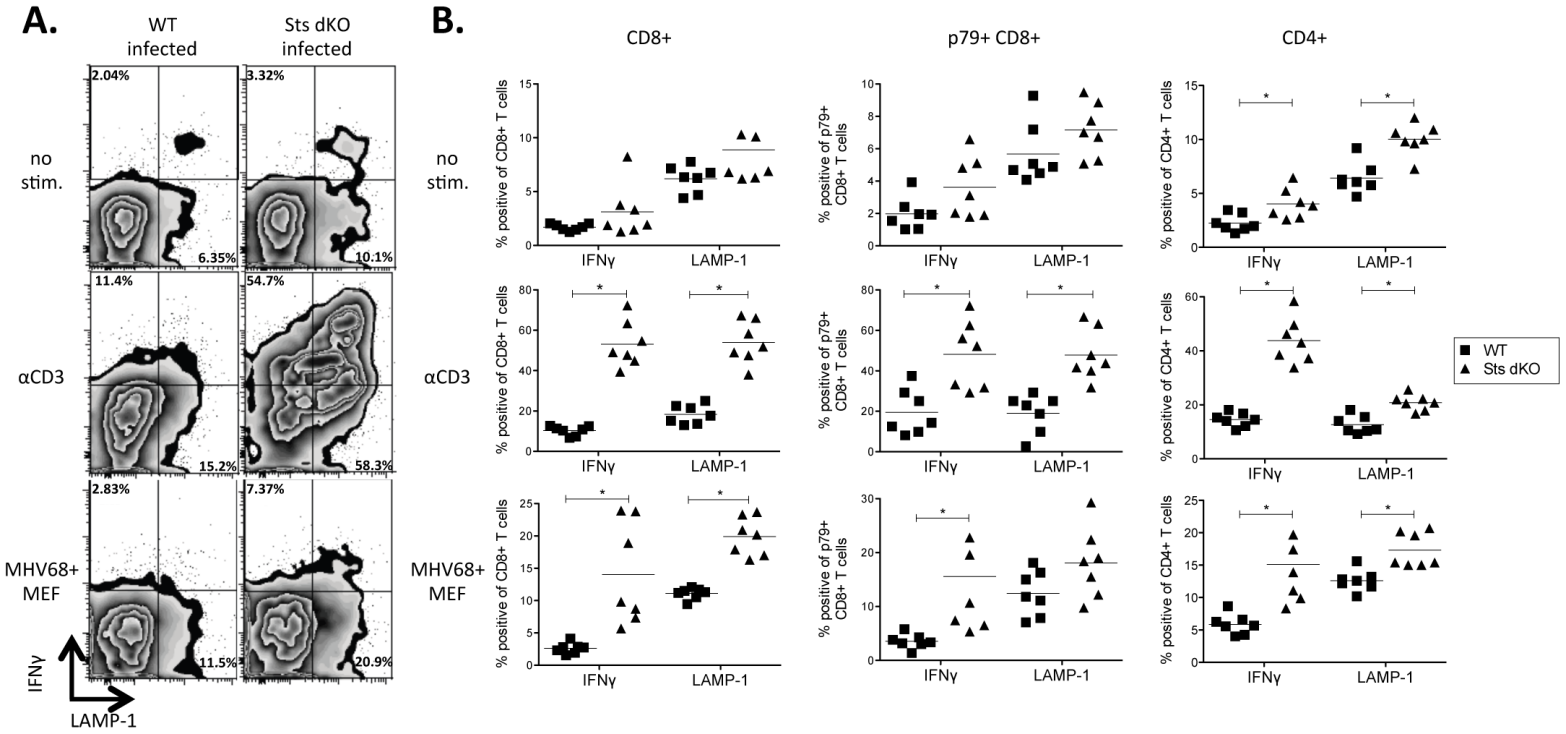
Sts dKO CD4^+^ and CD8^+^ T cells have increased effector responses to infected cells. Sts dKO and C57BL/6 WT mice were infected 1000 PFU of MHV68 by the intranasal route and spleens were harvested 28 dpi. Stimulations were performed as described in Figure 1 with the addition of αLAMP1, followed by costaining for CD4, CD8, and p79 tetramers. (A) Representative flow plots of LAMP-1 and IFNγ expression on CD8^+^ T cells are shown for each genotype and culture condition. (B-D) Scatter plot of the percentage of the indicated T cell subsets positive for IFNγ or LAMP-1. Axes were adjusted to better illustrate differences within each experimental condition. Symbols represent data from individual mice. * = p<.05, ** = p<.01, *** = p<.001. ****  = p<.0001.

We next sought to determine the effector function of both CD4^+^ and CD8^+^ T cell subsets by examining IFNγ production and LAMP1 surface staining. LAMP1 marks perforin-containing granules docked at the plasma membrane as an indicator of degranulation. Splenocytes isolated from naïve or infected WT and Sts dKO animals 28 dpi were incubated overnight with 1 µg/ml αCD3 antibody or cocultured with infected MEFs in the presence of monensin. As illustrated in the flow plots of Figure 2A and summarized in the scatter plots of Figure 2B, untreated CD8^+^ T cells from Sts dKO animals had a slight elevation in LAMP1 production compared to their WT counterparts. αCD3 stimulation induced significantly more IFNγ and LAMP1 in the CD8^+^ T cells from infected Sts dKO mice (Figure 2B, left column). Interestingly, Sts dKO CD8^+^ T cells from infected mice had significantly more IFNγ and surface LAMP1 upon coculture of splenocytes with MHV68-infected MEFs relative to WT CD8^+^ T cells. We next investigated the responses of CD8^+^ T cells specific to the viral p79/ORF61 epitope (Figure 2B, middle column). Without stimulation, the frequency of p79-tetramer+ virus-specific T cells was comparable between infected Sts dKO and WT mice (Table 1), and we observed no difference in IFNγ production or LAMP1. However, TCR stimulation with αCD3 antibody or infected MEFs induced significantly greater IFNγ and LAMP1 in the virus-specific T cells lacking Sts-1 and Sts-2 (Figure 2B, middle column).

**Table 1:** Immune response in the lungs during acute infection.

	Mean (SD) %^a^	
Cell type	WT	Sts dKO
CD4+ T cells	11.68 (0.95)	15.92 (7.53)
Effector CD4+ T cells	28.2 (6.13)	37.04 (16.11)
CD8+ T cells	18.2 (7.19)	15.2 (5.49)
Effector CD8+ T cells	58.79 (17.38)	66.4 (12.34)
p56 tetramer+ T cells	8.78 (4.96)	9.28 (6.21)
B cells	13.31 (4.46)	12.31 (3.49)
Activated B cells	4.21 (1.65)	3.97 (0.79)

a , The data shown are the percentage +/− standard deviation for each subset derived from FACS analysis of individual infected mice.

CD4^+^ T cells play an important role in controlling MHV68 [32]. There was a striking increase in IFNγ production and LAMP1 staining in CD4^+^ T cells from Sts dKO infected mice compared to CD4^+^ T cells from WT infected mice prior to stimulation (Figure 2B, right column). TCR complex stimulation with αCD3 antibody induced significantly more IFNγ and LAMP1 in Sts dKO CD4^+^ T cells than in WT cells, and this heightened response was also observed upon stimulation by virus-infected MEFs (Figure 2B, right column). Collectively, the absence of Sts-1 and Sts-2 enhances the T cell response to direct TCR stimulation by CD3 or MHV68-infected cell antigens.

### The absence of Sts-1 and Sts-2 does not alter lytic replication in vivo

**Figure 3:**
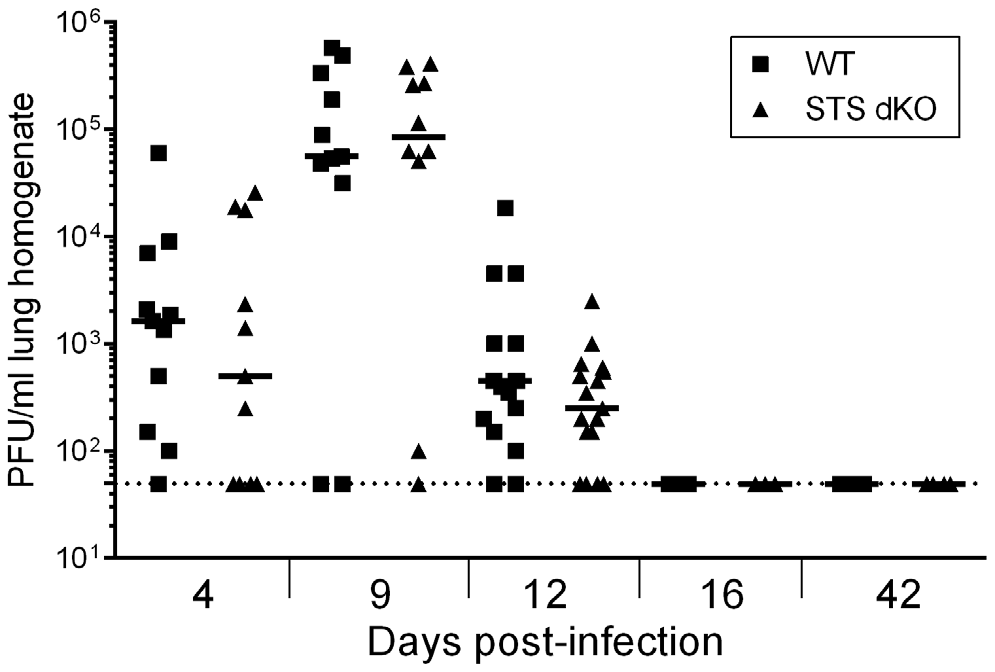
Sts dKO T cells do not alter the kinetics or levels of acute replication in the lungs. Sts dKO and C57BL/6 WT mice were infected 1000 PFU of MHV68 by the intranasal route. Lungs were harvested and disrupted at the indicated timepoints to quantitate levels of pre-formed infectious virus. Bar indicates median of log_10_ transformed data; dashed line indicates limit of detection. 3–8 animals are included per replicate experiment, n = 3 for 4 dpi, n = 2 for 9 dpi, n = 2 for 12 dpi, and n = 1 for 16 and 42 dpi; no significant differences were found based on Mann-Whitney non-parametric t-tests.

The loss of Sts-1 and Sts-2 leads to greater antiviral cytokine production and degranulation by T cells from infected mice upon stimulation in culture (Figure 2). However, the role of Sts-1 and Sts-2 in virus replication is unknown. We examined whether loss of Sts-1 and Sts-2 impacted MHV68 replication *in vitro* by examining replication in primary MEFs and primary bone marrow-derived macrophages (BMDMs) prepared from WT and Sts dKO mice. We observed no difference in virus production in either MEFs infected at a low MOI (0.05) or high MOI (5.0) or in BMDMs at a MOI of 10 (Figure S1). Next, we tested whether heightened effector functions would impact acute MHV68 infection *in vivo* by measuring virus replication in the lungs of infected Sts dKO and WT mice at multiple timepoints. The course of acute replication in Sts dKO and WT mice proceeded with similar kinetics and peaked at similar levels at 9 dpi (Figure 3). Examination of the immune response at 12 dpi revealed no differences in B cell or T cell subsets recruited to the lungs, including no change in the percentage of virus-specific CD8^+^ T cells as evidenced by staining for the early immunodominant epitope of ORF6 with the p56 tetramer (Table 1). Virus was cleared from the lungs by 16 dpi and remained undetectable at later time points of chronic infection in both genotypes (Figure 3). We also observed no reduction in MHV68 replication in spleens after intraperitonal infection (Figure S2A). These findings indicate that Sts-1 and Sts-2 do not influence acute virus replication.

Previous studies have demonstrated that the transfer of virus-specific T cells provides protection against primary MHV68 infection [31], [35] and prevents the expansion of MHV68-immortalized B cells in immunocompromised mice [57], [58]. We tested if the transfer of T cells from infected Sts dKO or WT mice could confer protection against primary infection in naïve mice. Donor T cells were enriched from the splenocytes of infected Sts dKO mice and WT mice 28 dpi. Naïve WT mice received 1×10^5^, 1×10^6^, or 1×10^7^ donor T cells by retro-orbital transfer 24 hours prior to MHV68 infection (Figure S3A). We observed a dose-responsive trend in the reduction of virus replication at 6 dpi with a significant nearly one-log decrease in viral burden upon the transfer of 1×10^7^ T cells (Figure S3B). However, both Sts dKO T cells and WT T cells generated similar levels of protection, indicating that in these experimental conditions, WT T cells were as potent as Sts dKO T cells in conferring protection against MHV68 replication *in vivo*.

### Immune response is minimally altered in Sts dKO mice

We next monitored if knockout of Sts-1 and Sts-2 alters the T cell profile or the generation of CD62L^lo^/CD44^hi^ effector T cells over a timecourse of chronic infection. We observed fewer CD4^+^ T cells at 28 dpi in the infected Sts dKO mice, but the levels increased to that of WT mice by 6 weeks post infection. Sts dKO mice had reduced levels of CD4^+^ effector T cells at 16 dpi, but had equivalent amounts at 28 dpi, and more at 6 weeks post infection (Table 2). CD8^+^ T cell populations that were lower at 16 dpi in the absence of Sts-1 and Sts-2 returned to WT levels by 28 dpi. On the other hand, effector CD8^+^ T cells were significantly increased at 28 dpi in the Sts dKO mice. We observed no difference in virus-specific p79^+^ CD8^+^ T cells at early or late stages of latency. The sustained expansion of Vβ4 CD8^+^ T cells is an immunological hallmark of MHV68 infection of C57Bl/6 mice [59]. The production of IFNγ from Vβ4 CD8+ T cells represses reactivation from peritoneal macrophages [51]. Interestingly, there was an enhanced Vβ4^+^ CD8^+^ T cell response at 28 dpi that returned to WT levels by 6 weeks post infection in the absence of Sts-1 and Sts-2.

**Table 2:** T cell subsets in the spleens of infected mice.

	Mean (SD) %, a											
	CD4+ T cells		Effector CD4+ T cells		CD8+ T cells		Effector CD8+ T cells		p79 tetramer+ T cells		Vβ4+ CD8+ T cells	
dpi	WT	Sts dKO	WT	Sts dKO	WT	Sts dKO	WT	Sts dKO	WT	Sts dKO	WT	Sts dKO
12	11.81 (2.08)	10.38 (3.19)	6.04 (1.09)	7.82 (0.98)	12.54 (2.34)	9.85 (1.45)	14.0 (6.28)	21.87 (5.84)	1.34 (0.76)	1.75 (0.95)	nd	nd
16	17.8 (3.65)	15.37 (1.3)	33.6 (1.3)	26.8 (2.46)	12.6 (1.41)	7.16 (1.23)	25.6 (9.12)	32.57 (8.20)	nd	nd	nd	nd
28	16.87 (3.93)	12.5 (3.44)*	57.07 (3.94)	60.97 (2.66)	17.48 (4.81)	18.53 (4.92)	45.76 (9.34)	58.69 (6.71)*	3.97 (1.84)	4.18 (3.87)	29.27 (9.96)	38.95 (8.51)*
45–57	19.05 (3.83)	16.6 (3.18)	20.9 (4.81)	28.63 (2.24)	23.8 (2.57)	19.48 (5.61)	33.5 (9.14)	28.84 (9.01)	nd	nd	28.84 (12.97)	28.55 (12.87)

a , The data shown are the percentage +/− standard deviation for each subset derived from FACS analysis of individual infected mice. *, significant difference (p<0.05) between infected WT and STS dKO mice.

In addition to being the primary targets for latency establishment in the spleen, B cells also mediate a humoral response to MHV68 infection [60], [61]. We examined if the lack of Sts-1 and Sts-2 altered B cell frequency and responses to infection. Levels of B cells in Sts dKO animals were higher at 16 dpi, but not at later time points (Table 3). Activated B cells in Sts dKO animals were higher at 28 dpi but returned to WT levels by 6 weeks post infection. Germinal center B cells typically undergo rapid expansion that peaks approximately two weeks after MHV68 infection [62]. This expansion was observed for both WT and Sts dKO infected mice, but the frequency of germinal center B cells was lower in Sts dKO mice compared to WT mice. Taken together, the loss of Sts-1 and Sts-2 led to transient differences in T cell and B cell responses at early and late periods of chronic infection.

**Table 3:** B cell subsets in the spleens of infected mice.

	Mean (SD) %, a					
	B cells		Activated B cells		Germinal Center B cells	
dpi	WT	Sts dKO	WT	Sts dKO	WT	Sts dKO
12	39.27 (2.55)	45.57 (2.38)*	nd	nd	nd	nd
16	55.17 (2.83)	56.3 (6.76)	nd	nd	2.70 (1.14)	2.21 (1.14)
28	43.13 (5.49)	43.26 (5.24)	3.28 (0.22)	3.82 (0.48)*	1.18 (0.45)	1.43 (0.47)
45–57	22.1 (2.33)	24.6 (1.82)	11.38 (0.63)	10.68 (0.38)	2.38 (0.50)	1.70 (0.09)*

a , The data shown are the percentage +/− standard deviation for each subset derived from FACS analysis of individual infected mice. *, significant difference (p<0.05) between infected WT and STS dKO mice.

### Sts-1 and Sts-2 do not impact latency or reactivation from latency

**Figure 4:**
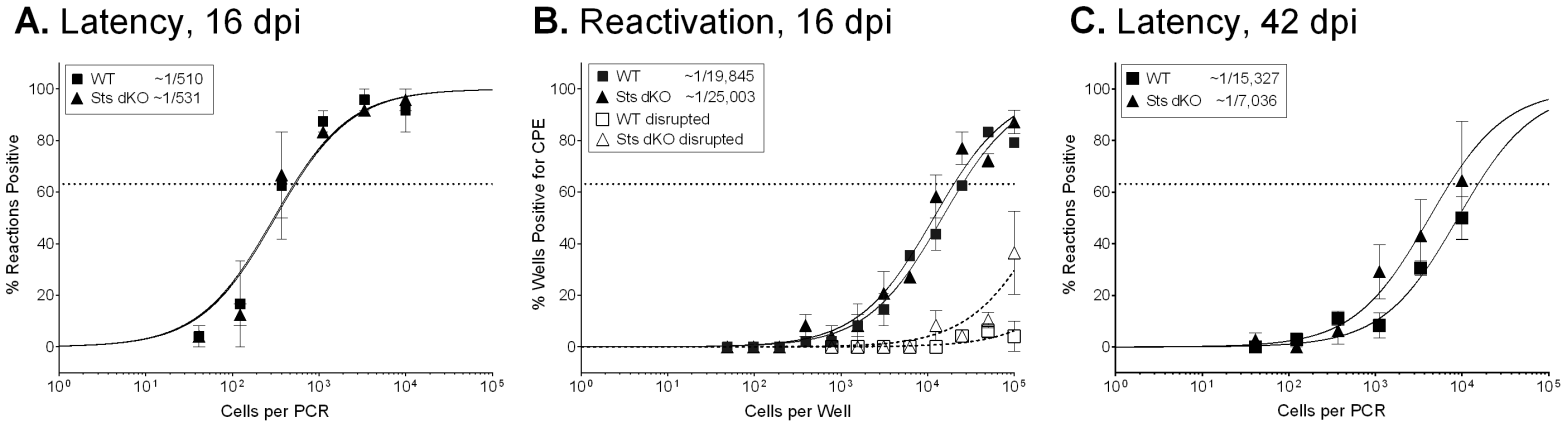
Viral latency and reactivation from latency are unchanged in Sts dKO animals. Sts dKO and C57BL/6 WT mice were infected with 1000 PFU of MHV68 by the intranasal route and single cell suspensions of the spleens were prepared at 16 dpi. (A) Frequency of splenocytes harboring viral genomes determined by limiting dilution nested PCR assay. (B) Frequency of splenocytes reactivting virus determined by a limiting dilution ex vivo reactivation assay. (C) Frequency of splenocytes harboring viral genomes at 45-57. For all limiting-dilution assays, curve fit lines were derived from nonlinear regression analysis, and symbols represent the mean percentage of wells positive for virus (viral DNA or CPE) ± the standard error of the mean. The dotted line represents 63.2%, from which the frequency of viral genome-positive cells or the frequency of cells reactivating virus was calculated based on the Poisson distribution. Graphs represent 3 independent experiments of 3–4 mice.

Sts dKO mice did not exhibit substained alterations in effector T cell responses or the B cell profile between two and six weeks after infection, suggesting a normal course of virus colonization of the spleen. However, Sts-1 and Sts-2 might play intrinsic roles in the latent cell reservoirs that influence establishment of latency and reactivation from latency. Next, we sought to examine whether Sts-1 and Sts-2 influence viral latency in the spleen at early and late time points during chronic infection. To examine changes in latency, we determined the frequency of MHV68-genome positive splenocytes by a limiting dilution nested PCR assay. At 16 dpi we observed no significant difference in the levels of viral latency in the spleen (Figure 4A). Previous studies found that mice lacking CD8^+^ T cells, IFNγ, or perforin exhibit increased reactivation [9], [29], [63]. To examine the role of Sts-1 and Sts-2 in reactivation from latency at 16 dpi, we performed a limiting dilution plating assay wherein serial dilutions of intact splenocytes are co-cultured with a monolayer of mouse embryonic fibroblasts that are monitored for cytopathic effect at two and three weeks post infection. There was no change in the frequency of splenocytes undergoing reactivation upon explant from infected mice in the absence of Sts-1 and Sts-2 as compared to WT mice (Figure 4B). We sought to increase the number of virus-specific T cells present in the explant co-culture reactivation assay by the addition of enriched T cells (70% purity) from previously infected mice at a 5∶1 ratio with splenocytes from WT infected mice at 16 dpi. Co-culture with an enriched population of T cells from either Sts dKO or WT infected mice 28 dpi did not alter levels of reactivation (Figure S2B). Because T cells achieve control of latent gammaherpesviruses in the peritoneal exudate macrophages through the secretion of IFNγ [29], we also enriched T cells from Sts dKO or WT infected mice prior to co-culture at a 5∶1 or 25∶1 ratio with peritoneal exudate cells (PECs) from infected mice. The hyperresponsive T cells from infected Sts dKO mice did not alter levels of PEC reactivation (Figure S2C). Lastly, we examined long-term latency and found that levels of latency in Sts dKO mice remained similar to WT levels up to 57 dpi (Figure 4C). These data indicate that the lack of Sts-1 and Sts-2 does not alter pathogenesis during acute infection or at early or late times during chronic infection.

## Discussion

In this study we examined how the loss of Sts-1 and Sts-2 influences viral pathogenesis, with a particular interest in whether Sts dKO T cells that exhibit hyper-responsiveness to TCR engagement influence the host immune response and control of gammaherpesvirus pathogenesis. When isolated from MHV68-infected mice, T cells lacking the Sts proteins displayed striking increases (relative to wild-type T cells) in effector responses in response to antibody or viral antigen stimulation. Similarly, *Sts-1* ^-/-^ */2* ^-/-^ mice had altered splenic T cell responses. However, the absence of Sts-1 and Sts-2 did not impact acute replication, latency establishment or maintenance, and did not reduce reactivation from latency.

Unexpectedly, the enhanced response to infection by the Sts dKO T cells did not impart greater host control. During MHV68 infection of C57BL/6 mice, perforin is critical in maintaining latency in the spleen [29] and controlling viral recrudescence in the lung in the absence of CD4 T cells [54]. When stimulated *ex vivo* with virally infected cells, Sts dKO T cells expressed both increased levels of surface LAMP1, indicative of degranulation and perforin secretion, and increased levels of the effector cytokine IFNγ. However, we detected no change in the initial levels of latency established in the spleen or reactivation from splenic latency in Sts dKO mice. Analysis of latency during later time points revealed that Sts dKO mice maintained MHV68 at levels similar to WT. In addition, incubation of explanted splenocytes or peritoneal macrophages with purified T cells from infected mice did not alter levels of reactivation. Taken together, the straightforward interpretation is that T cells enhanced by the removal of these negative TCR regulatory molecules are not functionally significant in the control of gammaherpesvirus latency and reactivation during a normal course of infection in an immunocompetent animal. However, it must also be noted that CD4^+^ T cells and CD8^+^ T cells have antagonistic functions during infection. For instance, upon CD4^+^ T cell depletion in mice infected with MHV68 and the ensuing viral recrudescence in the lungs, Molloy et al. [31] observed that CD8 T cells secrete the immunosuppressive cytokine IL-10. The administration of antibodies against the IL-10 receptor restored host control of reactivation. Therefore, it is possible that analysis of the individual role of CD4^+^ T or CD8^+^ T cell subsets might better reveal specific functions of helper or cytotoxic T cells whose functions have been altered by removal of Sts-1 and Sts-2. In this context, it is also important to note that we have observed recently a significant increase in the frequency of peripheral CD4^+^ regulatory T cells that are normally thought to act in an immunosuppressive capacity (Carpino, unpublished observations). Thus, the absence of an *in vivo* impact in immunocompetent mice reported here may represent a net neutral outcome from a more complex array of opposing T cell effector functions that will only be revealed by targeted antibody depletions or reconstitution with specific T cell subsets.

Examination of early acute infection by MHV68 revealed that the hypersensitive Sts dKO T cells did not aid in control or clearance of the virus in the lungs of infected mice. There was no difference in the infiltration of B cell or T cells, including p56 tetramer+ virus-specific T cells, into the lungs at 12 dpi. Previous studies demonstrate that the transfer of T cells specific to MHV68 epitopes reduced initial viral replication and reduced latency and long-term maintenance [31], [35]. Although we observed a decrease in lytic virus in the lungs after transfer of high numbers of T cells, Sts dKO T cells did not provide increased levels of protection in our adoptive transfer experiments. Based on the fact that CD8^+^ T cells specific to the immunodominant p79 epitope/ORF61 comprised approximately 4% of the total CD8^+^ T cell population (Table 2), we surmise that only a small percentage of the T cells that were transferred were specific to viral epitopes. Further enrichment of virus-specific T cells in combination with an increased dose of infectious particles may reveal a protective effect of hyper-responsive Sts dKO T cells. Sehrawat et al. [35] recently reported the generation of TCR transgenic mice specific for lytic epitopes of MHV68; a source of mono-specific T cells would enhance adoptive transfer studies that seek to enhance T cell effector responses.

Restoration of immune surveillance via the adoptive transfer of T cells specific to both lytic and latent EBV antigens is a highly efficacious therapeutic intervention in the context of immune suppression [64]. Gammaherpesvirus-associated lymphoproliferative diseases, such as the EBV-associated nasopharyngeal carcinoma and post-transplant lymphoproliferative disease, express more immunogenic targets than latently infected B cells [65], and adoptive transfer of *in vitro*-expanded virus-specific T cells from an autologous or homologous source can efficiently control transplanted virus-infected tumor lines or PTLD [57], [66], [67]. However, adoptive T cell transfer is not always effective in eliminating EBV-associated malignancies [68]. T cell therapies primarily focus on the expansion of virus-specific CD8^+^ T cells, but CD4^+^ T cells have significant direct effector roles [69], [70] in addition to enhancing CD8^+^ T cell function [70], [71]. An additional challenge is that gammaherpesviruses may induce the T-cell suppressive cytokine IL-10 [72] or encode an IL-10 homologue (vIL-10) [73], [74]. In EBV+ PTLD, elevated IL-10 levels have been correlated with an increased failure to respond to adoptive T cell transfer [75]. Mechanisms that enhance T cell responses may substitute for CD4^+^ T cell help or counter inhibitory cytokines produced by the host to improve the efficacy of adoptive T cell transfer.

We conclude that the heightened Sts dKO T cell responses upon stimulation in culture do not confer increased immune control and do not induce pathology in immunocompetent mice infected with MHV68 over an extended timecourse. Interestingly, MHV68 infection and the lack of Sts-1 and Sts-2 have each been reported to promote the development of experimental autoimmune encephalomyelitis [47], [76]. Future adoptive T cell transfer studies will examine whether virus-specific Sts dKO CD8^+^ T cells can augment the control of gammaherpesvirus infections or clearance of lymphomas in immunodeficient hosts. More studies are warranted to ensure that Sts dKO T cells can be therapeutic without leading to autoimmune complications in the context of MHV68 and other infections.

## Supporting Information

Figure S1 **MHV68 replication in WT and Sts dKO cells.** (A) Murine embryonic fibroblasts cells (MEFs) were infected with MHV68 at a MOI 0.05 or 5. (B) Primary bone marrow-derived macrophages were infected with MHV68 at a MOI 10. At the indicated times post infection, cultures were freeze-thawed and titered on NIH 3T12 fibroblasts.
(TIF)

Figure S2 **Effect of Sts dKO on intraperitoneal infection and reactivation from peritoneal macrophages.** Mice were infected intraperitoneally with 1000 PFU of MHV68. (A) Viral titer was measured in the spleen 9 dpi. Bar indicates median of log_10_ transformed data and the dotted line marks the limit of detection. Data represents two experiments of 4–8 animals; no significant differences were found based on Mann-Whitney non-parametric t-test. (B) Splenocytes and (C) peritoneal exudate cells were harvested from ten WT mice 16 dpi and reactivation was measured by a limiting dilution *ex vivo* reactivation assay without T cells or with enriched T cells from Sts dKO or WT infected mice 28 dpi. The ratios of T cells to target cells are indicated in the legend.
(TIF)

Figure S3 **T cell transfer prior to infection reduces acute replication.** (A) Schematic of T cell transfer experiment. Sts dKO and C57/BL6 WT mice were infected 1000 PFU of MHV68 by the intranasal route and spleens were harvested 28 dpi. Naïve mice received phosphate buffered saline (PBS) or the indicated numbers of enriched T cells by retroorbital transfer one day prior to intranasal infection with 1000 PFU MHV68. (B) Lungs were harvested 6 dpi and pre-formed infectious virus was measured by plaque assays. Symbols represent individual animals; * =  p>0.05.
(TIF)

Methods S1 The file Methods S1.pdf contains additional information to the manuscript explaining materials and methods for the supporting information Figure S1, Figure S2, and Figure S3. It consists of 2 pages.
(PDF)
