# Supplementary figures and images for: Enhanced Response of T Cells from Murine Gammaherpesvirus 68-Infected Mice Lacking the Suppressor of T Cell Receptor Signaling Molecules Sts-1 and Sts-2
Source: PLoS One. 2014 Feb 28;9(2):e90196. doi: 10.1371/journal.pone.0090196 (PMC3938662; doi:10.1371/journal.pone.0090196)

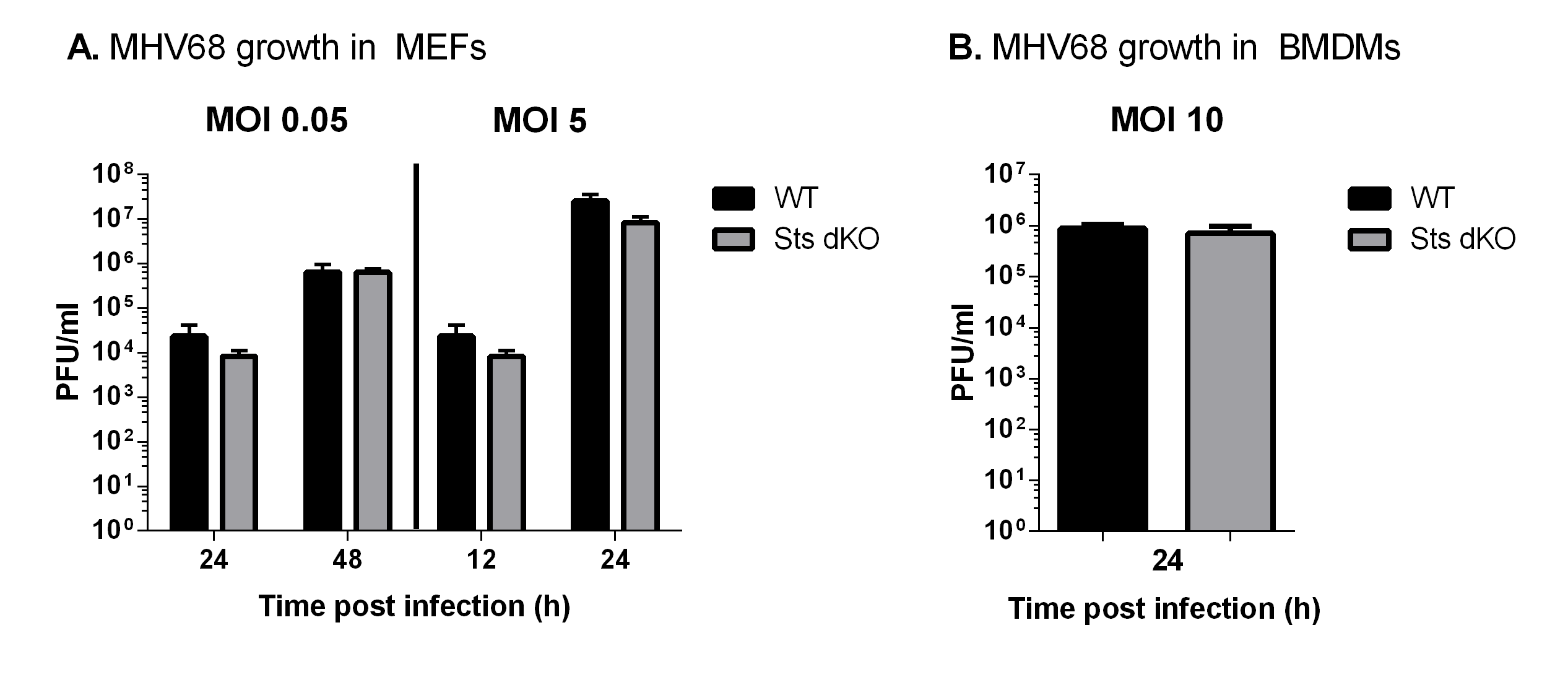

Supplement: Figure S1 — MHV68 replication in WT and Sts dKO cells. (A) Murine embryonic fibroblasts cells (MEFs) were infected with MHV68 at a MOI 0.05 or 5. (B) Primary bone marrow-derived macrophages were infected with MHV68 at a MOI 10. At the indicated times post infection, cultures were freeze-thawed and titered on NIH 3T12 fibroblasts. (TIF) [file pone.0090196.s001.tif]

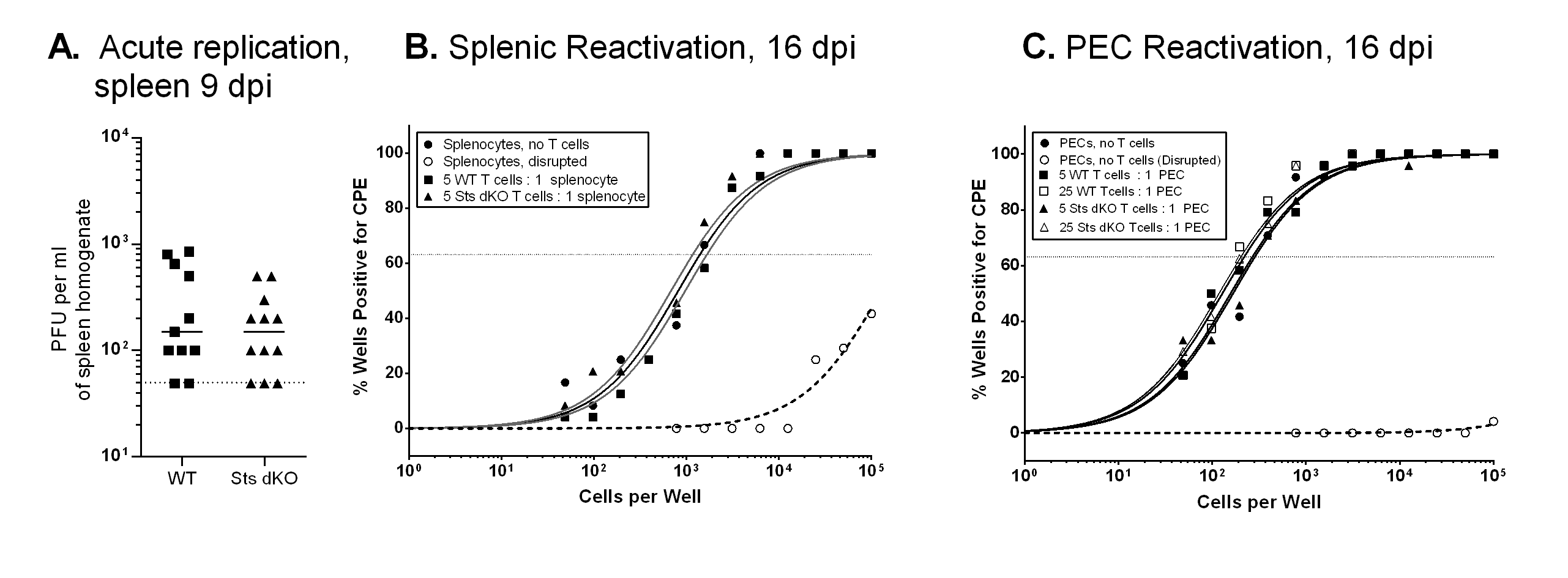

Supplement: Figure S2 — Effect of Sts dKO on intraperitoneal infection and reactivation from peritoneal macrophages. Mice were infected intraperitoneally with 1000 PFU of MHV68. (A) Viral titer was measured in the spleen 9 dpi. Bar indicates median of log10 transformed data and the dotted line marks the limit of detection. Data represents two experiments of 4–8 animals; no significant differences were found based on Mann-Whitney non-parametric t-test. (B) Splenocytes and (C) peritoneal exudate cells were harvested from ten WT mice 16 dpi and reactivation was measured by a limiting dilution ex vivo reactivation assay without T cells or with enriched T cells from Sts dKO or WT infected mice 28 dpi. The ratios of T cells to target cells are indicated in the legend. (TIF) [file pone.0090196.s002.tif]

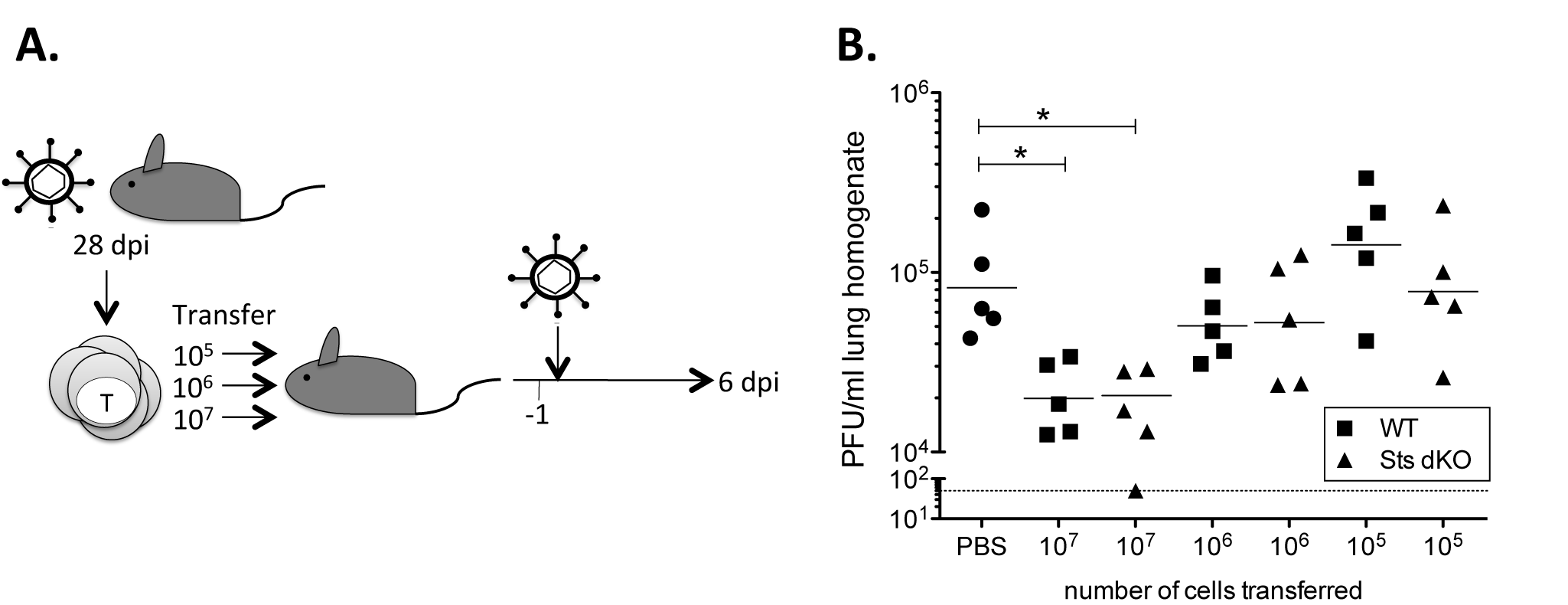

Supplement: Figure S3 — T cell transfer prior to infection reduces acute replication. (A) Schematic of T cell transfer experiment. Sts dKO and C57/BL6 WT mice were infected 1000 PFU of MHV68 by the intranasal route and spleens were harvested 28 dpi. Naïve mice received phosphate buffered saline (PBS) or the indicated numbers of enriched T cells by retroorbital transfer one day prior to intranasal infection with 1000 PFU MHV68. (B) Lungs were harvested 6 dpi and pre-formed infectious virus was measured by plaque assays. Symbols represent individual animals; * = p>0.05. (TIF) [file pone.0090196.s003.tif]
