# Supplementary material for: Enhanced Response of T Cells from Murine Gammaherpesvirus 68-Infected Mice Lacking the Suppressor of T Cell Receptor Signaling Molecules Sts-1 and Sts-2
Source: PLoS One. 2014 Feb 28;9(2):e90196. doi: 10.1371/journal.pone.0090196 (PMC3938662; doi:10.1371/journal.pone.0090196)
Supplement: Methods S1 — The file Methods S1.pdf contains additional information to the manuscript explaining materials and methods for the supporting information Figure S1, Figure S2, and Figure S3. It consists of 2 pages. (PDF) [file pone.0090196.s004.pdf]

Methods S1

Cell culture and virus infections

Primary MEFs were infected with MHV68-WT at a low (0.05) or high (5) MOI. Cells were infected for 1h, washed with PBS, then 8% CMEM was added. Bone marrow-derived macrophages (BMDMs) were generated by isolating bone marrow from 8-12 wk old mice from the femur and differentiation for 5d in DMEM with Glutamax (Life Technologies) containing 20% FBS and 30% L-supplement (BMM-Hi) in non-tissue culture treated plates. Cells were maintained in DMEM with Glutamax containing 10% FBS and 15% L-supplement (BMM-low) in non-tissue culture treated plates. BMDMs were infected with MHV68-eYFP at high MOI (10.0). At the indicated times post infection, cells were freeze-thawed and serial dilutions were titered as described for acute titers.

Pathogenesis

Mice were infected by intraperitoneal infection with 1000 PFU of MHV68 in 0.5 ml 10% CMEM, For acute titers, speens were harvested from mice at 9 dpi and titered as described above. At 16 dpi, peritoneal exudate cells were isolated by injecting the peritoneum with 10 ml of 8% CMEM, followed by shaking the abdomen and then removing the media with a syringe. For latency and reactivation assays, spleens and peritoneal exudate cells were isolated and analyzed by the limiting dilution assays described above. For T cell co-culture assays, enriched T cells were added at a 5:1 or 25:1 ratio to the splenocytes or peritoneal exudate cells in the explant reactivation plates.

T Cell Transfer

Sts-1/2 dKO and C57BL/6 mice were infected as described above. Spleens were removed 28 dpi, homogenized in CMEM, red blood cell lysed, and filtered through a 100 µM filter. T cells were isolated using positive selection with αCD90.2 microbeads (Miltenyi Biotech). Purity was analyzed by staining cells for CD3 and CD19 with flow cytometry, as described above. WT mice were anesthetized with 5% isofluorane via face mask, and 10^7, 10^6, and 10^5 purified Sts-1/2 dKO or C57BL/6 T cells were injected

retroorbitally in 5x C57BL/6 mice. 24 h later mice were infected with 1000 PFU WT MHV68 as described above. After 6 days, lungs were titered as described above. Bar represents geometric mean. *=$p<0.05$.
